# Diagnostic Significance of Cytokeratin 20 and p53 as Adjunct Immunocytological Markers in Urine Cytology to Detect Urothelial Carcinomas: A Systematic Review Study

**DOI:** 10.30699/IJP.2023.1972203.3004

**Published:** 2023-07-16

**Authors:** Evelyn Angel

**Affiliations:** Department of Pathology, Sree Mookambika Institute of Medical Sciences, Kulasekharam. The Tamilnadu Dr. M.G.R Medical University, India

**Keywords:** Cytokeratin 20, p53, Sensitivity, Specificity, Urine cytology, Urothelial carcinoma

## Abstract

The progression and recurrence of urothelial carcinoma (UC) are correlated with carcinoma *in situ* and urothelial dysplasia. It is frequently challenging to distinguish dysplasia and carcinoma *in situ* from reactive atypia only based on histological characteristics. In daily practices, 2 of the adjunct immunohistochemistry markers (cytokeratin 20 (CK20) and p53) are used in addition to the histology to diagnose carcinoma *in situ*. This is accomplished by combining histological research results with immunohistochemistry. This systematic review summarizes the current findings on the diagnostic significance of p53 and CK20 as adjunct markers to urine cytology in the detection of UC. A systematic search of the relevant literature was conducted using PubMed, Wiley Online Library, and ScienceDirect databases. After screening for the eligibility criteria, a total of 14 selected articles were reviewed. Data extraction included a total number of samples, specimen samples, type of cells, and outcome parameters (mainly sensitivity and specificity). Urine cytology alone had a sensitivity of 75%-85% and specificity of 66%-95%. CK20 with urine cytology staining showed improved sensitivity and specificity in the range of 77%-94% and 71%-100%, respectively; p53 immunostaining with urine cytology showed a sensitivity of 52%-86% and specificity of 80%-98%. The dual staining in combination with urine cytology showed comparatively higher sensitivity and specificity in the range of 70%-90% and 74%-100%, respectively. This was more evident for high-grade UC (HGUC). Overall, single or dual staining combined with urine cytology was effective in this detection and can be applied as an adjunct marker in urine cytology.

## Introduction

The neoplastic change of the urothelium (transitional cell) of the urinary bladder is the common reason for multiple bladder malignancies (1). The urothelium that lines the urinary bladder is a multi-layered epithelium with 3 cell zones: a basal cell layer, intermediate cell zone, and luminal or superficial cell layer (1). The neoplastic transformation in these cell lines is the common origin of transitional cell carcinoma or urothelial carcinoma (UC), a common type of bladder cancer (2). Globally, UC is the fifth most common type of cancer (3), characterized by a fast progressing rate and frequent recurrence rate. Different invasive techniques (such as cystoscopy, surgery, and biopsy) and noninvasive screening techniques have been widely used for the surveillance of urinary bladder cancer (4).

Among the noninvasive techniques, urine cytology, a technique developed by George Papanicolaou, is most widely used for the diagnosis and surveillance of UC. This technique involves the microscopic evaluation of exfoliated cells in urine to detect malignancies associated with the urinary tract, particularly the detection of high-grade UC (HGUC) (5). The principle of this technique works on the fact that tumor cells of the bladder are shed in the urine, where consequent necrosis and lysis of tumor cells release degraded proteins (such as keratin), which can be quantitatively measured (6). Noninvasive, low-cost, and high specificity are the main advantages of this technique (1). Mounting research evidence indicates the use of urine cytology in the screening of bladder cancer, particularly the differentiation of HGUC from LGUC and the follow-up of UC (7,8). 

Over the years, researchers have suggested multiple issues associated with the Papanicolaou technique, including a low sensitivity to detect low-grade urothelial neoplasms (1), lack of precision, and poor inter-and intra-observer variability have been a constant challenge associated with this technique (9,10). Besides, poor cytopathological practices (including variability in the collected urine samples, low or inadequate cellularity, degradation of the cells before the fixation, and untimely and improper preservation of the cellular material) led to variability in the morphological evaluation of the cells, thereby resulting in a non-uniform data on sensitivity, accuracy, and reproducibility of this technique. Thus, the clinical utility of this technique has remained a challenge (5). Few studies have associated urine cytology with a low sensitivity for bladder cancer detection, poor prediction of low-grade urothelial carcinoma, and poor distinction of atypical urothelial cells (AUCs) from UC. Besides, the poor abundance of neoplastic cells in most samples and the absence of a “tissue-like” structure remain major challenges in urine cytology (8). Urine cytology was found to be inefficient for the accurate detection of low-grade UC (LGUC) and AUCs (8).

Further, the infiltrative and aggressive nature of recurrent and high-grade neoplasm is an indicator of neoplastic changes in the urothelium and quicker progression to UC (11), demanding an immediate need to identify the biological markers for early detection and prognosis of the disease. Currently, despite the high specificity of urine cytology, the clinical utility of urine cytology to diagnose UC is limited due to its low sensitivity. Researchers have been continuously working to improve the sensitivity of urine cytology (8). Noninvasive tests or ancillary techniques on voided urine samples (such as immunocytochemical markers, including methylation markers, protein markers, and others) have been developed (4,12). In this context, different immunohistochemistry markers (including p53 (13) and cytokeratin 20(CK20) (14) have been reported to identify the neoplastic changes and progression of UC. The immunohistochemical profile of p53 and CK20 in urothelial dysplasia indicates the presence of abnormal cells in the urothelium (11). 

CK20 is a 40- to 70-kD cytoskeletal protein (also known as an intermediate filament) and is expressed in epithelial cells (15). It shows tissue-specific expression and is mainly involved in defining the structure of the tissues. Rarely expressed in normal urothelium, their altered expression is noted in the tumor cells, including bladder cancer cells (16). At the protein level using immunohistochemistry (17) and at the mRNA level using reverse transcriptase–polymerase chain reaction (RT-PCR) (18), the expression of CK20 has been reported in bladder tissues and exfoliated cells (7). The immunohistochemical staining of CK20 has been used to identify the nature of UCs, such as papillary Ucs of limited malignant potential (PUNLMP), LGUC, and HGUC (2). Thus, according to current research, urine CK20 might be a potential non-invasive biomarker for bladder cancer, particularly bladder UC (19). Further, the diagnostic accuracy of urine CK20 was observed to be better with the progression of tumor stage and grade. In the context of bladder cancer, p53 immunostaining has been used as a surrogate marker (20). The mutational status of the p53 gene and 2-fold higher expression in HGUC have been associated with the progression and recurrence of UC (21). In conjunction with urine cytology, p53 immunocytochemistry (ICC) has shown improved sensitivity and specificity in detecting malignant urothelial cells (22). Despite numerous studies, no single marker has been identified with sufficient sensitivity and specificity to be used as an adjunct to the detection of UC. Accordingly, the main aim of this comprehensive systematic review was to summarize the evidence on the use of CK20 and p53 alone or in combination as adjunct immunocytochemical markers in urine cytology to detect Ucs.

## Material and Methods

**Literature Search Strategy**

A systematic literature review was conducted to identify articles that assessed the diagnostic significance of CK20 and p53 as adjunct markers in urine cytology for the detection of Ucs. PubMed, Wiley Online Library and Science Direct databases were searched for relevant articles addressing the research question. The following search terms or MeSH words connected by 2 Boolean operators “OR” and “AND” were used: urothelial neoplasm OR urothelial carcinoma OR transitional cell carcinoma OR papillary urothelial neoplasm OR transitional cell neoplasm OR urinary bladder epithelial neoplasms OR bladder cancer OR low-grade urothelial carcinoma AND urine OR urine cytology OR urine liquid-based cytology OR urine sediments AND p53 OR TP53 OR cytokeratin 20 OR CK20 OR CK-20 AND immunocytochemistry OR adjunct marker. 

**Study Selection**

The PICO (population, intervention, comparison, and outcome) model was used to determine the inclusion and exclusion criteria for the systematic literature review. The inclusion criteria for the systematic literature review involved studies that investigated the diagnostic significance of p53 and CK20 immunostaining markers (intervention) in the urine samples (control) for the diagnosis of UCs or papillary UCs of low or high grade (outcome). According to Wong-You–Cheong JJ *et al.*^(1)^, the term urothelial tumors exhibit a broad spectrum of neoplasias ranging from benign papilloma through carcinoma *in situ* to invasive carcinoma; thus, articles mentioning UC or transitional cell carcinoma were also included. Following the screening, studies measuring the diagnostic outcomes/characteristics in terms of sensitivity and/or specificity of urine cytology, CK20, and/or p53 ICC alone or in combination with the urine samples to detect UC were reviewed. Further, the search was limited to original articles published in English. Review articles, comments, case reports, and letters to editors were excluded. Abstracts and full texts, wherever possible, were screened for the data (Figure 1). The details of the included studies are summarized in Tables 1 and 2. 

**Data Extraction**

From the selected studies, extracted data included type of study, sample size, sample specimen, type of cells, immunostaining markers other than p53 and CK20, and outcomes, including sensitivity, specificity, positive predictive value (PPV), and negative predictive value (NPV). 

**Figure:**
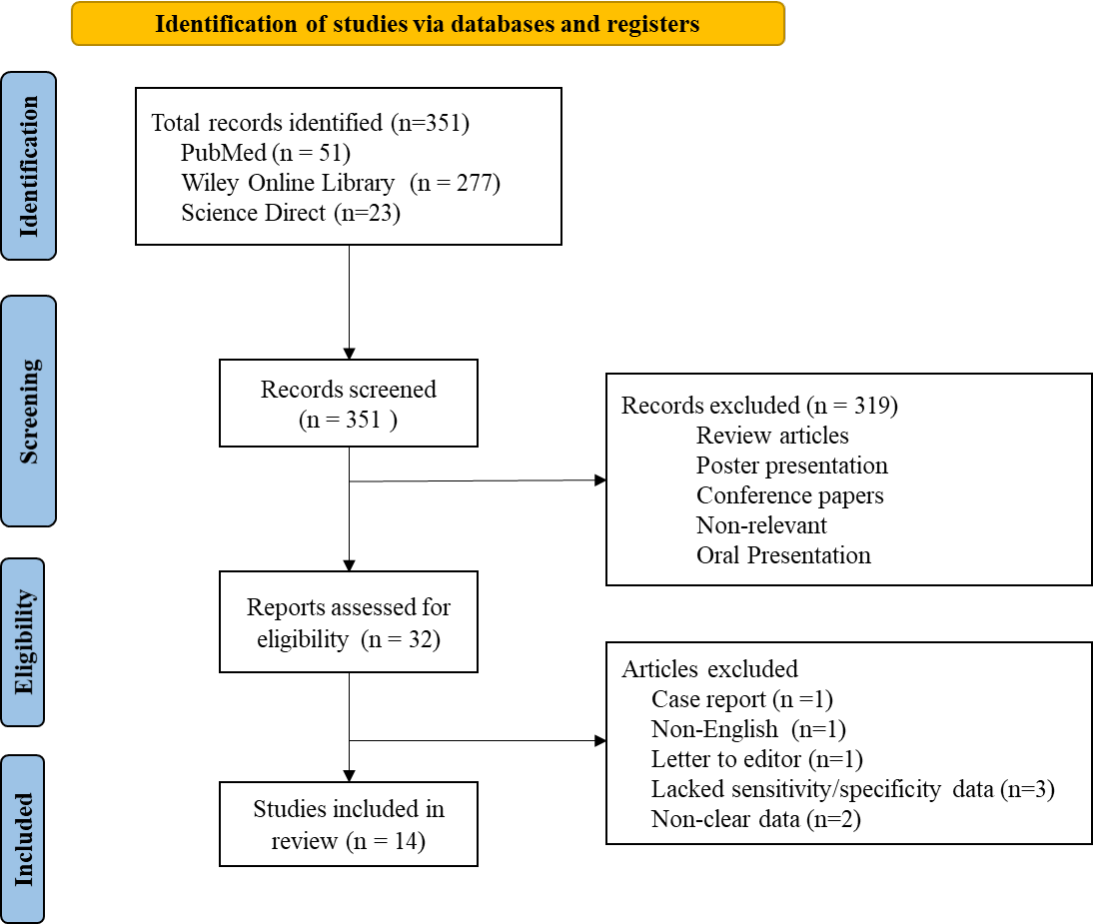


**Table:** 

Source/ References	Specimen	Total sample size	Type of cells/cases	Markers other than CK 20	Outcomes				
CK20					**Sensitivity**	**Specificity**	**PPV**	**NPV**	**Overall accuracy**
Wadhwa *et al.* (2017)	Fresh voided-urine samples	150	urothelial neoplasia: 42 (High grade: 14; low-grade and low malignantpotential: 28); non-neoplastic lesions: 20		Ucyt (all cases): 83.3%, CK20: 88.1%: Ucyt+CK20: 90.5%; Ucyt (HGUC): 100%, CK20: 100%: Ucyt+CK20: 100%; Ucyt (LGUC): 75.0%, CK20: 82.1%: Ucyt+CK20: 64.3%;	Ucyt (all cases): 65.0%, CK20: 95.0%: Ucyt+CK20: 95.0%; Ucyt (HGUC): 65.0%, CK20: 95%: Ucyt+CK20: 90%; Ucyt (LGUC): 65%, CK20: 95.0%: Ucyt+CK20: 90.0%;	Ucyt (all cases): 83.3%, CK20: 97.4%: Ucyt+CK20: 97.4%; Ucyt (HGUC): 66.7%, CK20: 93.3%: Ucyt+CK20: 87.5%; Ucyt (LGUC): 75%, CK20: 95.8%: Ucyt+CK20: 90.0%;	Ucyt (all cases): 65.0%, CK20: 79.2%: Ucyt+CK20: 82.6%; Ucyt (HGUC): 100%, CK20: 100%: Ucyt+CK20: 100%; Ucyt (LGUC): 65%, CK20: 79.2%: Ucyt+CK20: 64.3%;	
Srivastava *et al.* (2012)	Archived Papanicolaou stained urine cytology smears	59	urinary bladder carcinoma: 42 (HGUC:19; LGUC: 23); non-neoplasm (control): 17	NMP22	Ucyt: 76.2%, CK20: 70.4%: Ucyt+CK20: 66.7%	Ucyt: 76.5%, CK20: 71.4%: Ucyt+CK20: 71.4%	Ucyt: 94.1%, CK20: 90.5%: Ucyt+CK20: 90.0%	Ucyt: 76.5%, CK20: 38.5%: Ucyt+CK20: 35.7%	
Soyeur *et al.* (2009)	Urine samples	90	Control (non-neoplasm): 36; LGUC: 31; HGUC: 23		Ucyt: 75.9%, CK20: 70.4%: Ucyt+CK20: 77.8%	Ucyt: 66.7%, CK20: 83.3%: Ucyt+CK20: 66.7%	Ucyt: 77.3%, CK20: 86.3%: Ucyt+CK20: 77.7%	Ucyt: 88.9%, CK20: 65.2%: Ucyt+Ck20: 66.7%	Ucyt: 72.2%, CK20: 75.5%: Ucyt+CK20: 73.3%
Bhatia et al. (2007)	Papanicolaou-stained slide	14	Benign cytology:14; atypical cytology:5		86%	100%			
Morsi *et al. *(2006)	Mid-stream urine samples	50	Transitional cell carcinoma: 35; Squamous cell carcinoma: 15	CK19	Ucyt: 50%: Ucyt + CK20: 80.4%	Ucyt: 100%; Ucyt+CK20: 78%	NA	NA	
Melissourgos *et al.* (2005)	Spontaneously voided urine samples	232	TCC: 144 (62 at diagnosis and 82 in follow-up); 88: non-TCC		Ucyt: 54.2%; CK20: 65.3%	Ucyt: 86.4%; CK20: 90.9%	Ucyt: 86.7%; CK20: 92.2%	Ucyt: 53.5%; CK20: 61.5%	
Lin *et al.* (2001)	Archived urine slides	77	atypical urine cytology and positive follow-up biopsy		94.40%	80.50%			
Golijanin *et al. *(2000)	Voided urine samples fixed on cytocentrifuge slides	174	hematuria or irritative voiding symptoms: 80; follow-up cases after resection of bladder tumors: 94		81.60%	77.00%			80%
p53				Markers other than p53					
Brisuda *et al.* (2019)	Cells block from voided urine samples	152	Urothelial carcinoma: 77; multiple benign urological conditions: 75	Ki-67, MCM2 and MCM5	For LGUC: p53: 12%; Ucyt+p53: 52%; For HGUC: p53: 75%; Ucyt+p53: 86.5%	For LGUC: p53: 93.3%; Ucyt+p53: 80%; For HGUC: p53: 75%; Ucyt+p53: 86.5%	NA	LGUC: p53: 76.1%; Ucyt+p53:83.3%	
Courtade‐Saïdi* et al.* (2016)	Urine samples on CytoSpin slides	196	low-grade UC (ALG), suspicious for high-grade UC (SHG) and high-grade UC (HG)		Ucyt (all cases): 86.4%; Ucyt+p53: 81.3%; Ucyt (ALG): 54.2%; Ucyt+p53: 68.8%; Ucyt (SHG): 86.7%; Ucyt+p53: 73.1%; Ucyt (HG): 92.5%; Ucyt+p53: 89.8%	Ucyt (all cases): 76.7%; Ucyt+p53: 93.2%; Ucyt (ALG): 88.0%; Ucyt+p53: 96.4%; Ucyt (SHG): 83.5%; Ucyt+p53: 96.4; Ucyt (HG): 97.1%; Ucyt+p53: 98.2%	Ucyt (all cases): 82.6%; Ucyt+p53: 94%; Ucyt (ALG): 59.1%; Ucyt+p53: 84.6%; Ucyt (SHG): 66.7%; Ucyt+p53: 90.5%; Ucyt (HG): 96.1%; Ucyt+p53: 97.8%	Ucyt (all cases): 81.5%; Ucyt+p53: 79.1%; Ucyt (ALG): 85.7%; Ucyt+p53: 91.5%; Ucyt (SHG): 94.3%; Ucyt+p53: 88.5%; Ucyt (HG): 94.3%; Ucyt+p53: 91.5%	

**Table:** 

Source/ References	Samples	Total sample size	Type of cells	markers other than CK20 and p53	Outcomes			
					**Sensitivity**	**Specificity**	**PPV**	**NPV**
Yi-Choi *et al. *(2021)	LBC using the ThinPrep	61	Negative: 19, atypical: 20; suspicious for HGUC: 16; LGUN: 2; HGUC: 4	NA	UC (all cases): 36.1%; LGUC: 29.4%; HGUC: 38.6%; UC+DIC (all cases): 70.5%; LGUC: 52.9%; HGUC: 77.3%			
Lenos* et al. *(2017)	ThinPrep smear urine cytology samples	125	Samples with hypocellularity (11); non-malignant (20); malignant (58) and atypical (36); Histological type: HGUC: 52; LGUC: 27; Negative: 35	NA	UCyt: 73.4%; DIC: 83.5%; UCyt with DIC: 91.1%,	UCyt: 100%; DIC: 74.3%; UCyt with DIC: 74.3%	UCyt: 100%; DIC: 88%; UCyt with DIC: 88.9%	UCyt: 62.5%; DIC: 66.7%; UCyt with DIC: 78.8%
Kim and Yoo (2017)	Urine samples stored in PreservCyt®solution	63	Malignant, highly suspicious, suspicious, and atypical	NA	Overall: 89%	Overall: 100%	Overall: 100%	Overall: 78%
Arville* et al. *(2013)	Paraffin-embedded urine cell blocks	102	Negative: 29; atypical:56; malignant: 17	CD44	Overall: 88.1%; without low-grade carcinoma: 94.6%	Overall: 78.3%; without low-grade carcinoma: 78.3%	Overall: 74%; without low-grade carcinoma: 72.9%	Overall: 90.4%; without low-grade carcinoma: 96%

## Results

**Literature Overview**

The systematic search of the literature is presented in Figure 1. Briefly, the search yielded a total of 351 articles from PubMed (n=51), Wiley Online Library (n=277), and Science Direct (n=23) databases. After the initial screening (which included the screening of titles and abstracts), 329 articles (including reviews, poster presentations, oral presentations, conference papers, and non-relevant articles) were removed. Then, the remaining 22 articles were screened for inclusion and exclusion criteria, and 8 articles were removed due to lack of clarity in data, lack of sensitivity and specificity data, non-English articles, letters to the editor, and case reports. Finally, a total of 14 articles comprising 1,545 samples were included in the systematic review. Of the 14 articles, 8 articles were related to CK20 ICC, 2 articles to p53 ICC, and 4 articles to dual staining (p53 and CK20) in urine samples. 

**CK20 as an Adjunct to Urine Cytology**

CK20, as a potential adjunct marker to urine cytology for the diagnosis of UC, has been tested by few studies. In the present study, a total of 8 studies assessing CK20 ICC in the urine samples were reviewed. An extensive test was done to assess the clinical utility of CK20 immunostaining as an adjunct to urine cytology in voided urine samples from 150 subjects with urothelial neoplasia of high-grade, low-grade, and low malignant, and non-neoplastic cells. All types of UCs were detected with higher sensitivity (90%), specificity (95%), PPV (97%), NPV (82%), and positive likelihood ratio (18) when the combination of urine cytology and Ck20 was used. However, the accuracy parameters were compromised when the combination was used to differentiate HGUC and LGUC. The combination of uCyt and CK20 had higher sensitivity (100% vs. 64.3%), NPV (100% vs. 64.3%), and positive likelihood ratio (10 vs. 6.43) to detect HGUC than LGUC. The specificity (90%) of the combination was similar for low-grade and high-grade tumors. According to the author, the CK20 test reduced the rate of undetectable exfoliated cells in patients with LGUC from 57.1% to 10.7%. The combination of urine cytology and CK20 immunostaining showed higher specificity and a positive likelihood ratio. 

A similar comparative study was performed by Soyuer *et al. *(24). The study included urine samples from 54 bladder cancer patients with primary or recurrent LGUC and HGUC. The sensitivity of urine cytology and CK20 is limited when used alone, with sensitivities of 75.9% and 70.4%, respectively; however, when combined, the sensitivity of urine cytology and CK20 improves to 77.8%.On the other hand, and the combination did not have any significant influence on the specificity, which remained low at 66.7%. In terms of the percentage of cells detected, the combination of urine cytology with CK20 detected 70.9% of low-grade tumors and 86.9% of high-grade tumors. Another comparative study (25) showed superior sensitivity, specificity, and reliability parameters of CK20 ICC over urine cytology. CK20 immunohistology was reported to have higher sensitivity (63.5% vs. 54.2%), specificity (90.9% vs. 86.4%), PPV (92.2% vs. 86.7%), and NPV (61.5% vs. 53.5%). The CK20 staining was also sensitive to detect different stages of bladder tumor. The CK20 immunostaining assay was found to be more sensitive to detecting stage pT1 (81.8% vs. 59.1%), grade 2 (76.2% vs. 61.9%), and grade 3 (82.1% vs. 67.9%) transitional cell carcinoma. The assay was also effective in detecting primary and recurrent tumors, despite its low sensitivity (~61%-68%). 

Srivastava *et al. *(26) performed CK20 immunostaining on archived Papanicolaou-stained urine cytology smears of bladder carcinoma. The sensitivity of CK20 immunostaining was 70.4%, and the specificity was 71.4%. The PPV was 90.5%, and the NPV was 38.5%. The combination of cytology and CK20 was found to have an overall sensitivity of 66.7% and specificity of 71.4%. The PPV was 90.0%, and the NPV was 35.7%. According to the author, when comparing urine cytology with CK20 immunostaining, there was no improvement in the sensitivity (76.2% vs. 70.4%) and specificity (76.5% vs. 71.4%) of CK20. A retrospective study (27) supported the use of the urine cytology in conjunction with CK20 to detect both low- and high-risk UCs. The authors used archived urine slides to test the potential of CK20 as an adjunct marker to detect AUCsof bladder carcinoma. With a threshold value of 5%, 27.3% of patients with atypical urine cytology and 94% of patients with atypical urine cytology and positive follow-up biopsy were positive for CK20. The authors reported high sensitivity (94.4%) and specificity (80%).

Bhatia *et al. *(28) looked at the utility of CK20 immunostaining in 14 unequivocal cases of UC. The test was highly sensitive (86%), with positive CK20 immunostaining observed in 12 out of 14 cases of UC, as well as highly specific (100%), as revealed by negative CK20 stains in all the cases with benign urine cytology. The authors found all the cells of atypical urine cytology positive for CK20. Golijanin*et al. *(29) found that CK20 ICC was effective in detecting tumors in 50% (n=87) of patients, with a sensitivity of 81.6%, specificity of 77%, and accuracy of 80%. Further, sensitivity varied with tumor grade, with a higher sensitivity of 93% and 92% for grades 2 and 3 bladder cancer, respectively, and a lower sensitivity of 56.5% for grade 1 bladder cancer. Further, the false negatives in low-grade and high-grade tumors were low in the range from 4% to 13.7%. This study advocated the use of CK20 to detect low-grade/stage bladder cancer. Further, in another study (30), based on receiver operating characteristic (ROC) curve analysis, an area under the curve (AUC) of 86.9% for CK20 was determined. In 50 urine samples from transitional cell carcinoma and squamous cell carcinoma patients, CK20 immunostaining improved the sensitivity from 50% to 80% at the cost of specificity that reduced from 100% to 78%. 

**P53 as an Adjunct to Urine Cytology**

In the present study, 2 studies relevant to immunocytochemical staining of p53 in urine samples to detect urothelial malignancies were reviewed. Among these, Brisuda *et al. *(31) used cell blocks, while Courtade-Saïdi *et al. *(32) used CytoSpin slides for p53immunocytochemical staining. In the study by Brisuda *et al. *(31), p53 (along with other markers, including MCM2, MCM5, and Ki-67) in combination with urine cytology was used to detect LGUC and HGUC. In this retrospective study, p53 immunostaining in combination with urine cytology reported improved sensitivity from 12% to 52% and NPV from 76.1% to 83.3% to detect LGUC while, regarding HGUC, improved sensitivity from 75% to 86.5% at the cost of low but acceptable specificity (93.3% vs. 80%). High sensitivity with low but acceptable specificity was observed to detect both HGUC and LGUC. The AUC value of 0.853 for p53 indicated the sensitivity and specificity of p53 to detect HGUC. According to the authors, the combination of ICC (p53) with urine cytology improved sensitivity at the cost of low specificity. 

Contrary to Brisuda *et al. *(31), Courtade-Saïdi *et al. *(32) concluded that p53 increased the specificity without reducing or penalizing the sensitivity. This particular study used dual markers (specifically p53 and Ki-67) in urinary cytology as possible adjunct markers in the detection of UC. With an optimal cutoff of 5%, p53 immunostaining was a significant method for identifying urinary cancer with cytological atypia that cannot exclude Low-grade urothelial carcinoma, suspicious for high-grade urothelial carcinoma, and High-grade urothelial carcinoma. Negative immunoreactivity for p53 in the case of normal cytology with a median percentage value (first to third quartile) of p53 was 0 (0-5), while positive immunoreactivity for p53 with Urothelial carcinomas was 30 (10-80). Urine cytology alone had a sensitivity and specificity of 86.4% and 76.7%, respectively. The combination of p53 with urine cytology did not bring a significant change to sensitivity (81.3%); however, specificity was significantly improved to 93.2%, and PPV was significantly improved from 82.6% to 94.0% to detect all cytology of UC. 

**P53 and CK20 Dual Staining in Conjunction with Urine Cytology**

Researchers have also attempted to improve the diagnostic accuracy of urine cytology by evaluation the dual immunocytochemical expression of marker proteins, namely, p53 and CK20. In the present review, studies involving dual (p53 and CK20) immunostaining to detect UC in urine samples were limited to 4. 

Arville *et al. *(33) used CK20 and p53 along with CD44 to distinguish HGUC from its mimics, including degenerative/reactive atypia. A total of 102 urine samples embedded in cell block paraffin (including 29 negative, 56 atypical, and 17 malignant/positive cytology samples) were used and divided into negative and positive follow-up groups. The detection rate for atypical cytology was low, ranging from 17.9% to 30.4%, while the detection rate for malignant tumors was high, ranging from 82% to 88%, for CK20, p53, and combination. The accuracy parameters were provided for the triple stain cocktail with an overall sensitivity of 88.1%, specificity of 78.3%, NPV of >90%, and PPV of 74%. The exclusion of low-grade urothelial carcinomas samples improved the sensitivity to 94.6%and NPV to 95.9%. Since the author did not ascertain the additional value of CD44, it was concluded that dual staining of p53 and CK20 was effective in distinguishing between benign and malignant cells.

The present review also included 2 retrospective studies (34,35). Kim and Yoo (34) included 63 urine samples stored in PreservCyt®solution with cytology of malignant, highly suspicious, suspicious, and atypical nature. Out of the samples tested, 34 showed positive results for CK20 and p53 immunostaining in highly suspicious, suspicious, and atypical cells, while samples negative for both CK20 and p53 were non-neoplasm cases. However, samples with low numbers of AUCs were not appropriate for immunostaining. The outcome parameter included a sensitivity of 89%, specificity of 100%, PPV of 100%, and NPV of 78%. The comparison of PPV between urine cytology alone and urine cytology combined with dual staining was 73% and 100%, respectively. Thus, this study advocated the use of dual staining for identification of the neoplastic cells.

Lenos *et al. *(35) compared urine cytology with a combination of urine cytology and dual ICC. The researcher classified liquid-based cytological samples (ThinPrep) of malignant (n=58), atypical (n=36), and negative (n=20) cytology into 2 broad categories based on the positive and negative immunostaining. Of the 58 malignant samples with LGUC(n=12) and HGUC (n=46), 89.6% were positive for at least 1 marker, 74% positive for CK20, 67.3% for p53, and 44.8% for both markers. In the case of atypical UC samples, 50% were positive for immunostaining. Among these, in atypical HGUC samples (n=6), 66% were positive for dual markers and 33% for CK20; however, in the case of atypical LGUC (11), 33% were positive for CK20 and p53 individually. Despite the low number of LGUC, the data indicated the detection of LGUC with either CK20 or p53. Besides, 31% of atypical with benign histology were also immunostained with either CK20 or p53. A comparison of the accuracy of cytologic diagnosis showed improved sensitivity from 73.4% to 91.1% and NPV from 62.5% to 78.8% with compromised specificity (100% to 74.3%) and PPV (100% to 88%) when Dual Immunocytochemistry was combined with the Urine cytology. 

Choi *et al. *(36)used Liquid-based cytology samples for immunocytochemical staining of p53 or/and CK20 on urine cytology samples diagnosed as negative (n=19), atypical (n=20), suspicious for HGUC (n=16), LGUN (n=2), and HGUC (n=4). Out of these, 8 out of 19 were negative, 13 out of 20 were atypical, 14 out of 16 suspicious for high-grade urothelial carcinoma and 4 out of 4 samples diagnosed as High grade urothelial carcinoma were positive for both CK20 and p53. Overall, the low sensitivity (36.1%) of urine cytology improved to 68.9% in combination with p53 and 70.5% in combination with p53 and CK20. Likewise, the sensitivity of urine cytology for low-grade and high-grade cancer improved from 29.4% to 52.9% and 38.6% to 77.3%, respectively, when combined with dual staining.

## Discussion

Growing evidence suggests a wide range of sensitivity and specificity of urine cytology in detecting various cytological features of bladder cancer or UC. According to Das *et al. *(37), urine cytology has a sensitivity of 89.8%, specificity of 69.23%, and diagnostic accuracy of 82.67% in detecting suspicious HGUC cells in midstream voided urine samples from patients with hematuria and UV neoplasms. Likewise, Abdullah (38) reported a sensitivity of 94.1% and a specificity of 26.3% in detecting malignant bladder cancer. On the contrary, Golijanin *et al. *(29) found voided urine cytology with high specificity, thus concluding the utility of urine cytology in determining the absence of a bladder tumor. Similarly, Morsi *et al. *(30) reported urine cytology with a sensitivity of 50% but 100% specificity, while Choi *et al. *(36) reported 29.4%-38.6% sensitivity of urine cytology to detect LGUC and HGUC. Further, some studies have reported that urine cytology has a sensitivity ranging from 75%-85% and a specificity ranging from 66%-95% for detecting bladder cancer (24,31,32)

ICC markers such as CK14 and CK20, ProEx C (39), and markers for cell proliferation such as Ki-67, p16INK4a, and p53 have been used as an objective immunostaining marker for distinguishing carcinoma *in situ* from non-neoplastic urothelium. Immuno-staining has its advantages: lower cost and high specificity as opposed to non-reproducibility, low sensitivity, and technical issues, which could result in false positives (30). To improve the accuracy of urine cytology, different adjunct tools (including ICC) using biomarkers (such as p53 and CK20) have been tested. According to Cheng *et al. *(6), IHC markers in urine cytology have a sensitivity of 60% specificity of 83% PPV of 77%, and NPV of 88%. Among included articles, a combination of CK20 and urine cytology showed a sensitivity of 77%-94% and specificity of 71%-100%, and a combination of p53 and urine cytology showed a sensitivity of 52-86% and specificity of 80-98%. The sensitivity and specificity of p53 and urine cytology combination were higher for HGUC in the range of 86%-92% and 86%-98% than for LGUC in the range of 52%-86% and 80%-88%, respectively. Likewise, for dual staining, the overall sensitivity of 70%-90%, specificity of 74%-100%, PPV of 88%-100%, and NPV of 78%-90% were estimated. In comparison, the sensitivity of dual staining was lower for LGUC than for HGUC (50% vs.73%). 

In a study of 89 patients with superficial UC, those who experienced recurrence during a 36-month follow-up period showed significantly higher overexpression of CK20 (63.4%) compared to those without recurrence (42.8%) in the context of CK20 ICC (40). According to the author, CK20 expression indicated differentiation of urothelial cells but did not support the use of CK20 as a predictor marker. The usefulness of CK20 as an adjunct marker for urine cytology has been proposed (27). In particular, the author showed that CK20 immunostaining could be conveniently performed on the same slide after routine morphological evaluation and is beneficial to triage atypical urine cytology into low- and high-risk categories for clinical follow-up (24) and also showed the superiority of ImmunoCyt/uCyt+, which is an im-munofluorescence-based assay, over the conventional urine cytology when combined with CK20. A higher sensitivity of 88.9% was observed for ImmunoCyt/uCyt+ with CK20 ICC compared to 77.8% for the conventional urine cytology-CK20 combination. The author also reported an improved sensitivity of 92.5% when combining these 3 noninvasive techniques (ImmunoCyt/uCyt+, conventional cytology, and CK20 ICC). According to this study, the low sensitivity in detecting low-grade tumors can be overcome by combining different noninvasive techniques. 

P53 has been quantified in the urine sediments of bladder cancer or bladder transitional cell carcinoma patients (41,42). Piaton *et al. *(43) identified the prognostic value of p53 in urine samples to detect UC. According to the authors, the uncertainty in the p53 staining in LGUC histological samples was due to the reasons such as modification of p53 in the tumor stage; thus, masking the antibody recognition, poor treatment, labeling protocols, and choice of antibody could be overcome by urine cytology. This study supported p53 immunostaining in urine-based samples to detect HGUC and LGUC (G1-2). This study lacked evaluation of accuracy parameters of urine cytology and therefore was not included in the present systematic review. However, this study paved a path to the use of p53 in ThinPrep LBC to overcome the limitations of p53 histological staining in UC tumors. In the present review, 2 studies performed p53 immunostaining to present the utility of p53 ICC as an adjunct to urine cytology. P53 immunostaining was found to be more sensitive and specific to detect UC cells (32,35,36). Brisuda *et al. *(31) and Courtade-Saïdi *et al. *(32) included LGUC or atypical cells to support the staining of p53 to detect LGUC. Brisuda *et al. *(31) showed the effectiveness of cell blocks used for ICC in combination with urine cytology to detect both HGUC and LGUC. The authors argued that a high NPV of immunocytology can benefit the follow‐up of LGUC. According to Brisuda *et al. *(31), p53 improved the sensitivity of urine cytology to detect HGUC, thereby supporting the use of p53 in the follow-up of high-grade tumors. It can be suggested that the higher or more efficient detection of p53 in HGUC may be attributable to a 2-fold higher mutation rate of the p53 gene compared to LGUC (21). Further, Brisuda *et al. *(31) reported that the use of additional markers (such as Minichromosome maintenance 5) p53 resulted in higher sensitivity and specificity in the detection of both LGUC and HGUC. 

CK20ICC has also been evaluated as an adjunctive marker for atypical cytology. In the context of CK20, the sensitivity was noted to be 65%, specificity was 90%, PPV was 86%, and NPV was 65% 6. The pitfall of CK20 ICC is the positivity of CK20 in benign umbrella cells. Thus, the nature of cells in the urine collection can affect the accuracy parameters (44). In urine cytology, Morsi *et al. *(30) showed positive cytoplasmic CK20 staining in the urine samples of TCC and an estimated 21.5 AU of CK20 in the urine samples from bladder cancer patients. In the present review study, a larger number of studies showed higher sensitivity and specificity of CK20 immunostaining over urine cytology to detect multiple bladder malignancies with a sensitivity ranging from 81.6%-83.3% and a specificity ranging from 70.4%-77%. Interestingly, Melissourgos *et al. *(25) reported higher specificity and positive predictive accuracy for the detection of bladder cancer. Collectively, 3 studies demonstrated sensitivities in the range of 65%-86% and specificities in the range of 86%-100% (24,25,28). Further, these studies also advocated the advantages of CK20 ICC over urine cytology in the detection of different stages of UC, including primary, recurrent, stage pT1, and grade 2/3 tumors. In the present review study, few studies were related to CK20 ICC in LGUC (23,24,26). However, their results showed contrasting specificity for the combination of uCyt and CK20. Wadhwa *et al. *(23) reported a specificity of 90% in the detection of all types of cancer, while Soyuer *et al. *(24) reported a low specificity of 66.7%, and Srivastava *et al. *(26) showed a specificity of 71.4%. Soyeur *et al. *(24) reported that the ImmunoCyt/uCyt+ (uCyt+™) test (Diagnocure), an immunocytological fluorescence assay along with CK20, produced better results in terms of sensitivity (82%) and specificity (80%) than conventional urine cytology. Based on the data, it can be inferred that Wadhwa *et al. *(23) validated the clinical utility of CK20 as a potential low-cost adjunct to urine cytology, particularly to diagnose LGUC. Thus, this marker can help to identify LGUC in patients. Lin *et al. *(27) also made a similar claim regarding the use of CK20 in detecting cancer in patients with atypical urine cytology. They also suggested that CK20 can be useful in distinguishing Carcinoma in situ from reactive atypia or dysplasia in biopsy samples. However, contrary to many findings, low sensitivity and low NPV results did not completely confirm the use of CK20 as an adjunct to urine cytology in the detection of UC (26). The author argued that the present result could be due to the use of the archived glass slides on which there was no adhesive. In addition, technical reasons (such as washing slides with xylene) could result in the loss of cells, which can hamper the results. Further, poor preservation of malignant cells and the presence of antigens can also interfere with the staining (26). The use of CK20 to distinguish atypical malignant cells from benign morphology was supported by Bhatia *et al. *(28). Thus, according to the author, CK20 could be the best bet to distinguish malignant from benign morphology. 

Overall, the reviewed articles showed mixed data on the accuracy parameters associated with dual staining. On the one hand, Lenos *et al. *(35) showed improved sensitivity (>90%) and NPV (>75%) comparable to Kim and Yoo (34) and Arville *et al. *(33) but with compromised specificity and PPV of dual immunostaining as an adjunct to urine cytology. Unlike Lenos, who reported a low specificity of 74% and PPV of 88%, Kim and Yoo showed improved specificity and PPV of uCyt with dual immunostaining. The sensitivity reported by Choi *et al. *(36) was comparatively lower than the sensitivity reported by other researchers. However, Choi *et al.* indicated improved sensitivity for LGUC, while Arville *et al. *(33) found improved sensitivity upon the removal of LGUC. The differences in the outcomes could likely be due to the differences in the diagnostic threshold and the type of sampling between the 2 studies. Further, in urine cytology, the positivity of p53 also depends on the cutoff values. For example, p53 positivity cutoff values of >5 and >10 positive cells for cell count and >5% for the positivity rate to detect UC were used by Choi *et al. *(36).

Regarding the parameters, the specificity of the stain can be affected by multiple reasons, including gross hematuria or inflammation; thus, to improve the specificity, a more specific tissue specimen will be required (30). In the present review, the comparison of data shows improved sensitivity upon the combination of dual immunostaining to urine cytology. The preservation of morphological structure during the process is crucial for the diagnosis (35). However, discordance in the number of cells (such as paucity of cells) can hamper the immunostaining (35). According to Soyuer *et al. *(24), urine cytology should be performed by a cytopathologist with expertise in pathology and urine cytology. This can help improve the sensitivity of the tests. From the conducted systematic review, it can be anticipated that antibodies of p53 and CK20 alone or in combination improved the detection, sensitivity, and specificity of urine cytology. However, a wide range of sensitivity also indicates higher levels of false negatives in the samples. Further, the technicalities of the test (such as pretreatment protocols, preparation of slides from urine samples, and ICC protocols) can also lead to different results. In this context, in immunocytochemical protocols, methanol is the best-suited fixation medium. However, different solutions (including 50% ethanol and acetone (32)) have been used as fixating agents. Further, differences can also be attributed to cytological preparations (such as liquid-based preparations and cell block preparations). The LBC technique involves a collection of cells on glass slides, and this method is reliable in the case of a smaller number of the cells. In addition, monolayer suspension of the cells, better fixation of the cells, and preserved nuclear details of cellular materials make them a better option for immunocytochemical analysis to detect malignant cells (46). Diagnostic accuracy of LBC in cytological samples other than urine, such as CSF (47), has also been shown. However, in the majority of the included studies, the cell block was the preferred technique due to its use for histology purposes; also, due to the paucity of the cells, cell-block preparation from urinary samples has remained challenging. Further, some discrimination in parameters can also arrive from the type of sampling. According to Wegelin *et al. *(45), differences in morphological characteristics in voided-urine and instrumented urine specimens could lead to inaccurate interpretations for the urothelial cell population (5). 

The present review study has some limitations. Not all the reported studies included the comparison of urine cytology with the combination of urine cytology and immunostaining. Comparison between immunohistochemical studies on tissue specimens and urine cytology in combination with ICC were not included in the study. Further, none of the reviewed studies conclude or suggest that these biomarkers should be used as a stand-alone method for the surveillance of UC. The accuracy parameters validate the use of CK20 and p53 as adjuncts to detect HGUC. Regarding the detection of LGUC, further experimentation is required. The present study showed promising results upon dual staining. The field of urinary biomarkers for the diagnosis of UC is rapidly evolving, and researchers are exploring the use of a wide range of biomarkers beyond p53 and CK20; therefore, prospective cohort studies with larger populations are required for the development and validation of the p53 and CK20 dual staining as an adjunct to the urine cytology in the detection of UC, particularly LGUC.

## Conclusion

Overall, studies investigating the use of single or dual staining in combination with the urine cytology have found this approach to be effective in the detection of UCs. The contribution of other markers (such as Minichromosome maintenance -5 and Nuclear matrix protein 22-2) has also been shown to improve the sensitivity and specificity of the urine cytology. Thus, more studies must be carried out to increase the significance of p53 and CK20 stains to detect malignant urothelial cells. Dual staining has significance as an adjunct marker in the urine cytology. P53 and CK20 have been shown to improve the sensitivity of the urine cytology as a stand-alone adjunct marker for diagnosis of UC; however, dual immunostaining, which combines use of two or more immunostains, such as p53 and CK20, has been found to provide even more diagnostic information and improve the accuracy of the urine cytology for detection of UC.

## Conflict of Interest

The authors declared no conflict of interest.

## References

[1] Wong-You-Cheong JJ, Woodward PJ, Manning MA, Sesterhenn IA (2006). Neoplasms of the urinary bladder: radiologic-pathologic correlation. Radiographics..

[2] Akhtar M, Rashid S, Gashir MB, Taha NM, Al Bozom I (2020). CK20 and CK5/6 immunohistochemical staining of urothelial neoplasms: A Perspective. Adv Urol..

[3] Bogen JP, Grzeschik J, Jakobsen J, Bähre A, Hock B, Kolmar H (2021). Treating Bladder Cancer: Engineering of Current and Next Generation Antibody-, Fusion Protein-, mRNA-, Cell- and Viral-Based Therapeutics. Frontiers in Oncology [Internet]..

[4] Calistri D, Casadio V, Bravaccini S, Zoli W, Amadori D (2012). Urinary biomarkers of non-muscle-invasive bladder cancer: current status and future potential. Expert Review of Anticancer Therapy..

[5] Barkan GA, Wojcik EM, Nayar R, Savic-Prince S, Quek ML, Kurtycz DF, Rosenthal DL (2016). The Paris system for reporting urinary cytology: the quest to develop a standardized terminology. Acta Cytol..

[6] Cheng L, Davidson DD, Maclennan GT, Williamson SR, Zhang S, Koch MO (2010). The origins of urothelial carcinoma. Expert Rev Anticancer Ther..

[7] Lokeshwar VB, Habuchi T, Grossman HB, Murphy WM, Hautmann SH, Hemstreet III GP (2005). Bladder tumor markers beyond cytology: International Consensus Panel on bladder tumor markers. Urology..

[8] Minasi S, Bosco D, Moretti B, Giangaspero F, Santoro A, Buttarelli FR (2021). Improvement of the Collection, Maintenance, and Analysis of Neoplastic Cells from Urine Specimens with the Use of CytoMatrix. Methods Protoc..

[9] Reid MD, Osunkoya AO, Siddiqui MT, Looney SW (2012). Accuracy of grading of urothelial carcinoma on urine cytology: an analysis of interobserver and intraobserver agreement. Int J Clin Experimentalpathol..

[10] Sugeeta SS, Sharma A, Ng K, Nayak A, Vasdev N (2021). Biomarkers in Bladder Cancer Surveillance. Frontiers in Surgery [Internet]..

[11] Mallofré C, Castillo M, Morente V, Solé M (2003). Immunohistochemical expression of CK20, p53, and Ki-67 as objective markers of urothelial dysplasia. Modernpathology..

[12] Budman LI, Kassouf W, Steinberg JR (2008). Biomarkers for detection and surveillance of bladder cancer. Canadian Urol Assoc J..

[13] Cina SJ, Lancaster-Weiss KJ, Lecksell K, Epstein JI (2001). Correlation of Ki-67 and p53 with the new World Health Organization/International Society of Urological Pathology classification system for urothelial neoplasia. Arch Pathol Lab Med..

[14] Alsheikh A, Mohamedali Z, Jones E, Masterson J, Gilks CB (2001). Comparison of the WHO/ISUP classification and cytokeratin 20 expression in predicting the behavior of low-grade papillary urothelial tumors. Mod Pathol..

[15] Jacob JT, Coulombe PA, Kwan R, Omary MB (2018). Types I and II keratin intermediate filaments. Cold Spring Harbour perspectives inbiology..

[16] Hong M, He G, Goh S, Low AW, Tay KJ, Lim TK (2021). Biomarkers for precision urothelial carcinoma diagnosis: Current approaches and the application of single-cell technologies. Cancers..

[17] Desai S, Dug Lim S, Jimenez RE, Chun T, Keane TE, McKenney JK (2000). Relationship of cytokeratin 20 and CD44 protein expression with WHO/ISUP grade in pTa and pT1 papillary urothelial neoplasia. Modern Pathology Off J U S Can Acad Pathol Inc..

[18] Rotem D, Cassel A, Lindenfeld N, Mecz Y, Sova Y, Resnick M (2000). Urinary cytokeratin 20 as a marker for transitional cell carcinoma. Europeanurology..

[19] Mi Y, Zhao Y, Shi F, Zhang M, Wang C, Liu X (2019). Diagnostic accuracy of urine cytokeratin 20 for bladder cancer: A meta-analysis. Asia Pac J Clin Oncol..

[20] Malats N, Bustos A, Nascimento CM, Fernandez F, Rivas M, Puente D (2005). P53 as a prognostic marker for bladder cancer: a meta-analysis and review. Lancet Oncol..

[21] Hodgson A, Xu B, Downes MR (2017). p53 immunohistochemistry in high-grade urothelial carcinoma of the bladder is prognostically significant. Histopathology..

[22] Enomoto K, Matsunaga T, Sofue T, Nakamura A, Hirakawa E, Ibuki E (2021). p53 expression in repair/reactive renal tubular cells: A potential pitfall leading to a false‐positive diagnosis of urine cytology. Cancer Medicine..

[23] Wadhwa N, Diwaker P, Lotha N, Arora VK, Singh N (2017). Cytokeratin 20 immunocytochemistry on urine sediments: a potential low-cost adjunct to cytology in the diagnosis of low-grade urothelial carcinoma. Cytopathol Off J Br Soc Clin Cytol..

[24] Soyuer I, Sofikerim M, Tokat F, Soyuer S, Ozturk F (2009). Which urine marker test provides more diagnostic value in conjunction with standard cytology- ImmunoCyt/uCyt+ or Cytokeratin 20 expression. Diagnosticpathology..

[25] Melissourgos ND, Kastrinakis NG, Skolarikos A, Pappa M, Vassilakis G, Gorgoulis VG (2005). Cytokeratin-20 immunocytology in voided urine exhibits greater sensitivity and reliability than standard cytology in the diagnosis of transitional cell carcinoma of the bladder. Urology..

[26] Srivastava R, Arora VK, Aggarwal S, Bhatia A, Singh N, Agrawal V (2012). Cytokeratin-20 immunocytochemistry in voided urine cytology and its comparison with nuclear matrix protein-22 and urine cytology in the detection of urothelial carcinoma. Diagnosticcytopathol..

[27] Lin S, Hirschowitz SL, Williams C, Shintako P, Said J, Rao JY (2001). Cytokeratin 20 as an immunocytochemical marker for detection of urothelial carcinoma in atypical cytology: preliminary retrospective study on archived urine slides. Cancer Detection and Prevention..

[28] Bhatia A, Dey P, Kumar Y, Gautam U, Kakkar N, Srinivasan R (2007). Expression of cytokeratin 20 in urine cytology smears: a potential marker for the detection of urothelial carcinoma. Cytopathology Off J Br Soc Clin Cytol..

[29] Golijanin D, Shapiro A, Pode D (2000). Immunostaining of cytokeratin 20 in cells from voided urine for detection of bladder cancer. The Journal of urology..

[30] Morsi MI, Youssef AI, Hassouna MEE, El-Sedafi AS, Ghazal AA, Zaher ER (2006). Telomerase activity, cytokeratin 20 and cytokeratin 19 in urine cells of bladder cancer patients. J Egypt Natl Cancer Inst..

[31] Brisuda A, Háček J, Čechová M, Škapa P, Babjuk M (2019). Diagnosis of urinary bladder urothelial carcinoma by immunocytology with p53, MCM5, MCM2 and Ki-67 antibodies using cell blocks derived from urine. Cytopathology Off J Br Soc Clin Cytol..

[32] Courtade-Saïdi M, Aziza J, d'Aure D, Bérard E, Evrard S, Basset C (2016). Immunocytochemical staining for p53 and Ki-67 helps to characterise urothelial cells in urine cytology. Cytopathol Off J Br Soc Clin Cytol..

[33] Arville B, O'Rourke E, Chung F, Amin M, Bose S (2013). Evaluation of a triple combination of cytokeratin 20, p53 and CD44 for improving detection of urothelial carcinoma in urine cytology specimens. Cytojournal..

[34] Kim HJ, Yoo JH (2017). Cytokeratin 20/p53 dual immunocytochemistry for improving the diagnostic accuracy of urine liquid-based cytology in the detection of urothelial neoplasm: A retrospective study. Cytojournal..

[35] Lenos M, Katafigiotis I, Vrettou K, Papaioannou D, Malta F, Trigka EA (2017). Evaluation of double immunocytochemical staining for CK20 and P53 as a potential adjunct to cytology for urothelial cancer diagnosis. Cytopathology Off J Br Soc Clin Cytol..

[36] Choi SY, Kim KH, Suh KS, Yeo MK (2020). Diagnostic significance of dual immunocytochemical staining of p53/cytokeratin20 on liquid-based urine cytology to detect urothelial carcinoma. Cytojournal..

[37] Das S, Roy Chowdhury AR, Basu S, Lath A (2019). A Study on Association of Urine Cytology as Reported by the Paris System with Histopathology and p53 Expression in Urinary Bladder Epithelial Neoplasms. J Clin Diagn Res..

[38] Abdullah LS (2013). The value of urine cytology in the diagnosis of bladder cancer. Cytopathological correlation. Saudi Med J..

[39] Moatamed NA, Rao JY, Alexanian S, Cobarrubias M, Levin M, Lu D (2013). ProEx C as an adjunct marker to improve cytological detection of urothelial carcinoma in urinary specimens. Cancer Cytopathol..

[40] Raica M, Zylis D, Cîmpean AM (2005). Cytokeratin 20, 34betaE12 and overexpression of HER-2/neu in urine cytology as predictors of recurrences in superficial urothelial carcinoma. Romanian Journal Morphol Embryol Rev Roum Morphol Embryol..

[41] Darabi M, Tayebi MN, Mahdavi R, Arab D (2006). p53 protein in serum and urine samples of patients with bladder transitional cell carcinoma and its overexpression in tumoral tissue. Urol J..

[42] El-Gamal EM, Gouida MS (2013). Quantitative changes in p53, Bcl-2 and apoptosis in blood and urine of bladder cancer patients. Clin Lab..

[43] Piaton E, Faÿnel J, Ruffion A, Lopez JG, Perrin P, Devonec M (2005). p53 immunodetection of liquid-based processed urinary samples helps to identify bladder tumours with a higher risk of progression. Br J Cancer..

[44] Sullivan PS, Chan JB, Levin MR, Rao J (2010). Urine cytology and adjunct markers for detection and surveillance of bladder cancer. Am J Translation Res..

[45] Wegelin O, Bartels DWM, Tromp E, Kuypers KC, Van Melick HH (2015). The effects of instrumentation on Urine Cytology and CK-20 Analysis for the Detection of Bladder Cancer. Urology..

[46] Sakamoto H, Takenaka M, Ushimaru K, Tanaka T (2012). Use of liquid-based cytology (LBC) and cell blocks from cell remnants for cytologic, immunohistochemical, and immunocytochemical diagnosis of malignancy. Open J Pathol [Internet]..

[47] Sioutopoulou DO, Kampas LI, Gerasimidou D, Valeri RM, Boukovinas I, Tsavdaridis D (2008). Diagnosis of metastatic tumors in cerebrospinal fluid samples using thin-layer cytology. Acta Cytol..

